# Does repetition equal more of the same? tie strength and thematic orientation in R&D networks

**DOI:** 10.1371/journal.pone.0303912

**Published:** 2024-05-23

**Authors:** Dima Yankova, Pablo D’Este, Mónica García-Melón

**Affiliations:** 1 INGENIO (CSIC-UPV), Universitat Politècnica de València, Valencia, Spain; 2 ANETI Lab, Corvinus Institute for Advanced Studies (CIAS), Corvinus University, Budapest, Hungary; Universidade Nova de Lisboa, PORTUGAL

## Abstract

Despite organizations’ documented tendency to repeat research collaborations with prior partners, scholarly understanding on the implications of recurring interactions for the content of the collaboration has been fairly limited. This paper investigates whether and under what conditions organizations use repeated research partnerships to explore new topics, as opposed to deepening their expertise in a single one (exploitation). The empirical analysis is based on the Spanish region of Valencia and its publicly funded R&D network. Employing lexical similarity to compare the topic and content of project abstracts, we find that strong ties are not always associated with the exploitation of the same topic. Yet, exploration is more likely when at least one of the partners mobilizes a network of distinct contacts and can access novel knowledge.

## 1. Introduction

The literature on inter-organizational networks has demonstrated organizations’ proclivity to repeat research interactions with prior partners, resulting in stronger ties and a reinforcement of existing network structures [1,2]. Empirical studies have documented this type of organizational inertia in partner selection within both national and international R&D networks [3–7]. Yet, the implications of repeated engagements for the content of the collaboration remain an issue of contested debate. Do organizations leverage repeated R&D ties to deepen their expertise in a particular thematic domain or could strong links also be associated with the exploration of different topics? The answer to these questions can shed light on the value of repeated ties for individual and collective performance.

Innovation scholars have argued that strong bonds between partners are subject to declining marginal benefits [8,9]. At first organizations accumulate gains from solidifying existing relationships, as transaction costs decrease [10] and the exchange of complex and tacit knowledge becomes easier, but beyond a certain threshold the learning potential for both parties may be exhausted [10–13]. Social embeddedness can begin to act as a filter for the entry of new knowledge and ideas, causing cognitive isolation and suboptimal innovative performance [14–16].

So far scholarly understanding on the role and functionality of repeated interactions has been constructed independently and with little consideration for the nature of ties [17,18]. Few studies have looked explicitly at interaction processes and the strategic decisions partners make when repeating research collaborations [19]. The goal of this paper is to shed light precisely on this issue, by comparing the topics of recurring collaborations. It investigates *whether* and *under what conditions* organizations use repeated engagements to explore new topics, as opposed to deepening their expertise in a single one.

This question is important for several reasons. If two organizations systematically exploit the same topic and tap into the same knowledge domain, their interactions will likely yield benefits at first, but hinder long-term innovative performance. If, however, subsequent collaborations begin to explore different topics, either because *a priori* the organizations involved possess a diverse internal repository of competencies and skills, or because they are capable of continuously sourcing novel knowledge through additional partnerships, the prospects of decreasing marginal benefits may weaken. Hence, the basic premise of this paper is that the relationship between strong ties and performance will be at least partially contingent on the nature of the exchange between partners, and their strategic orientation (exploration vs exploitation) in instances of repeated engagement. For the rest of the paper, we will use the term *exploitation* to denote persistent focus on the same topic in recurrent collaborations, while *exploration* refers to a shift in focus towards new topics, which differ from those addressed in the first instance of engagement between partners.

Disentangling the connection between the strength of ties and their thematic orientation merits scholarly attention also as a departure from the structuralist perspective, which dominates knowledge network studies, and which treats inter-organizational links as virtually homogeneous [19–21]. By qualifying strong ties based on their content, we can learn about the specific functions that seemingly identical types of relationships exercise in the context of inter-organizational networks [18,22,23].

To conduct the empirical analysis, we concentrate on the Spanish region of Valencia. We collected information on all R&D partnerships, formed between 2016 and 2022, which received public subsidy from one of the top two regional sources of innovation-related funding. The final dataset of 194 collaborative projects was used to map the local inter-organizational network and assess to what extent repeated engagements between partners in the 7 years of observations were associated with either topic exploration or exploitation. We also test how partners’ access to diverse knowledge and resources influenced the likelihood of them adopting one strategic approach over the other in subsequent collaborations. Given the rich literature on the benefits of degree centrality for learning, knowledge recombination and sustained innovative performance [24–27], well-connected actors may be more likely to explore new topics when re-engaging with the same partner.

This paper adds to a growing stream of literature that recognizes the importance of strong ties as a frequent phenomenon in interpersonal and interorganizational networks [28–30]. It aims to illuminate the interplay between the strength of a collaboration tie and its nature or thematic orientation (exploitative vs explorative). The contribution is thus twofold: first, we develop a theoretical argument to suggest that the relationship between repeated engagement and thematic orientation is fundamental for disentangling the effect of social cohesion on individual and collective performance. Though previous studies have demonstrated a clear link between network structural properties and actors’ performance [24,31–33], there is still relatively little understanding on the precise mechanisms which underlie this relationship [17,19,21]. Second, from a methodological perspective, our study applies recent advancements in machine learning and natural language processing (NLP) techniques to build a measure of thematic orientation that is based on the lexical similarity between project abstracts. Instances of NLP usage in the innovation and management literature are increasing [34–36], but they have concentrated primarily on patents’ textual data, whereas our goal is to showcase the potential of such methods to advance scholarly understanding of R&D networks and the value of inter-organizational linkages.

From a policy standpoint, our analysis is also highly relevant. In regional R&D networks, topic exploitation may be a desirable outcome if efforts are directed toward building competitive advantage in nascent or underexplored economic domains. Conversely, it can be highly undesirable if the network is stagnating, and policymakers are looking to branch out of existing development paths. Therefore, understanding how and under what conditions repeated engagement between regional partners is associated with either topic exploitation or exploration could help policymakers steer more effective network interventions.

The paper is structured as follows: section 2 lays the theoretical foundation of the study and relates it to the literature on inter-organizational networks and social capital. Section 3 introduces the characteristics of the dataset, and section 4 outlines our approach to operationalizing thematic orientation by building a measure of abstract similarity. Section 5 details the results of the analysis, while section 6 discusses their implications for theory and policy.

## 2. Theoretical background

### 2.1. The connection between the strength of ties and their thematic orientation (explorative vs exploitative)

A growing body of literature points to the importance of social embeddedness in driving the structural evolution of inter-organizational networks [37]. The formation of new partnerships between organizations is perceived in the context of their existing social structure and their history of prior ties [1,2]. Past engagement seems to impact the course of future cooperation in a path-dependent fashion, as former ties repeat themselves. This form of organizational inertia in partner selection has been observed across different types of networks. For instance, when analyzing the evolution of an industrial cluster in Italy, Lazzaretti & Capone found that the collaborative work experience developed between two actors is particularly influential in shaping tie formation during the cluster emergence phase, although less so in the development stage [38]. The presence of a previous relationship was also shown to influence SMEs’ partner selection in the process of consolidating a regional innovation network [4], while additional evidence suggests this effect intensifies during periods of crisis [39]. Similarly, in a study on university-industry research networks in the UK, D’Este & Iammarino noted the strong role played by prior joint experience, which the authors choose to conceptualize as a form of organizational rather than social proximity [5]. Balland et al. also observed a consistently stable effect of social embeddedness on inter-firm relations when analyzing the global video game industry [40]. Finally, several studies on EU-FP network collaboration patterns have highlighted the propensity of organizations, be they firms or public research bodies, to select familiar partners with whom they share a history of prior engagement [3,6,7].

The implications of strong ties for performance have been the subject of many empirical studies. Some highlight the benefits of strong bonds for fine-grained knowledge sharing, in line with Coleman’s theory on social capital [41]. Repeated engagements tend to engender “relational” trust between participating entities [42–44]. This can in turn reduce actors’ perception of expected opportunistic behavior, decrease transaction costs and ease the transfer of both *complex* and *tacit* knowledge [10–13,17,45,46]. On the other hand, strong ties between partners may also reinforce retention mechanisms and prevent the inflow or nonredundant information [15,16,47]. When organizational partners become so narrowly focused on a particular type of activity, a transition toward new developments becomes difficult, leading companies to display inferior economic performance [14].

Taking on board both perspectives, scholars have settled the relationship between strong ties and performance as an inverted U-shape. Organizations benefit from consolidating strong relationships up to a certain level, beyond which social embeddedness can act as a filter for the entry of new knowledge and perspectives, causing cognitive isolation and suboptimal innovative performance [8,9]. This situation is also known as “the proximity paradox”, since the same factors that drive actors to connect and exchange knowledge may also lead them to innovate less in the long run [48,49].

In this paper, we argue that the consequences of repeated collaborations for individual and collective performance cannot be fully disentangled without examining the content of ties and acknowledging that organizations may leverage repeated interactions for different purposes. In his seminal work on the strength of weak ties, Granovetter noted that “treating only the strength of ties ignores […] all the important issues involving their content”, and stressed that the relationship between strength and degree of specialization of ties deserves further analysis [50]. In addition, as pointed out by Reagans & McEvily many studies tend to *infer* knowledge transfer from the association between network structure–including tie strength–and performance, without directly examining the essence of the exchange [17,19,51].

In order to unpack this association, we employ the so-called “connectionist” perspective [20], which looks beyond the structural or topological properties of the network, and treats ties as conduits of knowledge and resource flow [18,22,23]. This approach acknowledges that seemingly identical types of links could exercise distinct functions and transmit varying kinds of resources. Furthermore, it recognizes that organizations in alliance networks are not simply “helpless targets of structural influence”, but active agents who make conscious decisions about the way they leverage strong bonds [19].

Take for instance the following scenario: if two organizations systematically tackle the same topic in multiple R&D collaborations, their interactions may follow the inverted U-shape scholars describe, whereby topic exploitation would at first yield positive outcomes, but if continued for too long would hamper innovative performance. In the process of exploiting the same topic multiple times, organizations are expected to tap into the same knowledge pool, building expertise at the start but eventually exhausting the recombination potential. If, however, subsequent collaborations begin to tackle different topics, either because *a priori* partners possess diverse internal repository of competencies, or because they are capable of sourcing those through third-party links, the graph of decreasing marginal benefits may take a different shape. At the very least, we can expect the threshold of redundancy, when the two partners have little learning space left, to become higher. Hence, the relationship between strong ties and performance is contingent on the content of the exchange between partners, and whether or not they choose to adopt an explorative or exploitative approach in repetitive engagements. This is not to suggest that one is inherently preferable than the other. Rather, our goal is to illustrate that structurally equivalent relations, in the form of strong bonds, can have very different consequences for knowledge exchange and learning depending on the content of the collaboration itself. To examine the heterogeneity of organizational approaches to repeated collaborations, we pose the following research question:

**Q1:** *To what extent do organizations leverage repeated collaborations to exploit the same topic multiple times or to explore new ones*?

### 2.2. Factors that moderate the relationship between strong ties and thematic orientation

The extent to which organizations use repeated collaborations to exploit prior topics or explore new ones may be influenced by their access to complementary knowledge and resources from third parties. Assuming that the knowledge repository of an entity is not static, forming alliances with a wide range of partners creates new pipelines for fresh ideas, perspectives, and information to flow [22,23]. This may in turn inspire greater diversification in the topics and content of repeated collaborations.

So far, multiple empirical studies have demonstrated that the size of a firm’s (*ego*) network, defined in terms of both direct and indirect contacts (*alters*), is positively associated with innovative output [24,27,31,52]. The theoretical framework, underlying these findings, assumes that well-connected organizations will have a more timely access to larger volumes of information through their established relationships. Yet, in line with the resource-based perspective [53], some researchers have argued it is not the sheer number of connections that matters, as much as the *diversity* of knowledge which can be sourced through direct relationships [31,54]. In other words, a focus on the composition of the ego’s first-order network, and more specifically the number of distinct partners, may be more appropriate. Assuming that each organization holds a unique set of assets and capabilities, direct relationships to multiple organizations may provide the best access to non-redundant knowledge and resources. In other words, we posit that the number of new first-order connections partners build before re-engaging with each other again may influence their propensity to explore new topics in a repeated exchange. To examine this issue further, we propose a second research question:

**Q2:** *To what extent does partners’ range of new connections to other organizations inspire new topic exploration in their repeated collaborations*?

## 3. Data collection and context

To investigate the relationship between repeated engagement and thematic orientation, we focus on an existing knowledge network formed by collaborative publicly funded R&D projects in the Spanish region of Valencia. The choice of a regional level network is appropriate given the widespread consensus that knowledge sharing tends to be highly concentrated and it mostly takes place in dense, local networks with rich social capital [55–57]. We extracted data on all awarded projects from the official grant resolution records of two regional organizations: the Valencian Institute for Business Competitiveness (IVACE) and the Valencian Innovation Agency (AVI). IVACE was established in 1984 and its mission is geared toward assisting regional SMEs in increasing their competitiveness and overall innovative capacity. AVI, on the other hand, was created more recently in 2018, specifically for the purpose of managing the innovation strategy of Valencia and improving the regional productive model. Together, these two organizations manage approximately 75% of the 1.6 billion Euros that the regional government has designated for the implementation of the local innovation strategy [58]. Thus, we can be fairly confident that by concentrating on AVI and IVACE, we are capturing a substantial amount of the publicly subsidized R&D collaboration network in the region.

According to public records, in the period 2016–2022 the two organizations funded a total of 220 collaborative R&D projects, under three lines of action: “R&D in cooperation” (IVACE), “Strategic projects in cooperation” (AVI) and “Consolidation of the business value chain” (AVI). While the programs managed by each organization have certain differences in eligibility criteria, they share a common purpose–to enhance downstream R&D cooperation between regional actors and to support the creation of new products, processes, or services through one of two types of projects: (1) industrial research and design or (2) experimental development. All three programs open calls on an annual basis, and none places restrictions with regards to research themes. Average project duration is between one and two years. The calls by IVACE are open to private companies only, while those managed by AVI allow for all type of regional actors, including universities, research centers, technological institutes, and even non-profits, to participate. The mean subsidy per project is 87000 EUR for IVACE, and 475000 EUR for AVI. Hence, we are *de facto* accounting for projects of different size and membership structure (firm-firm, firm-university, etc.).

**Fig 1:**
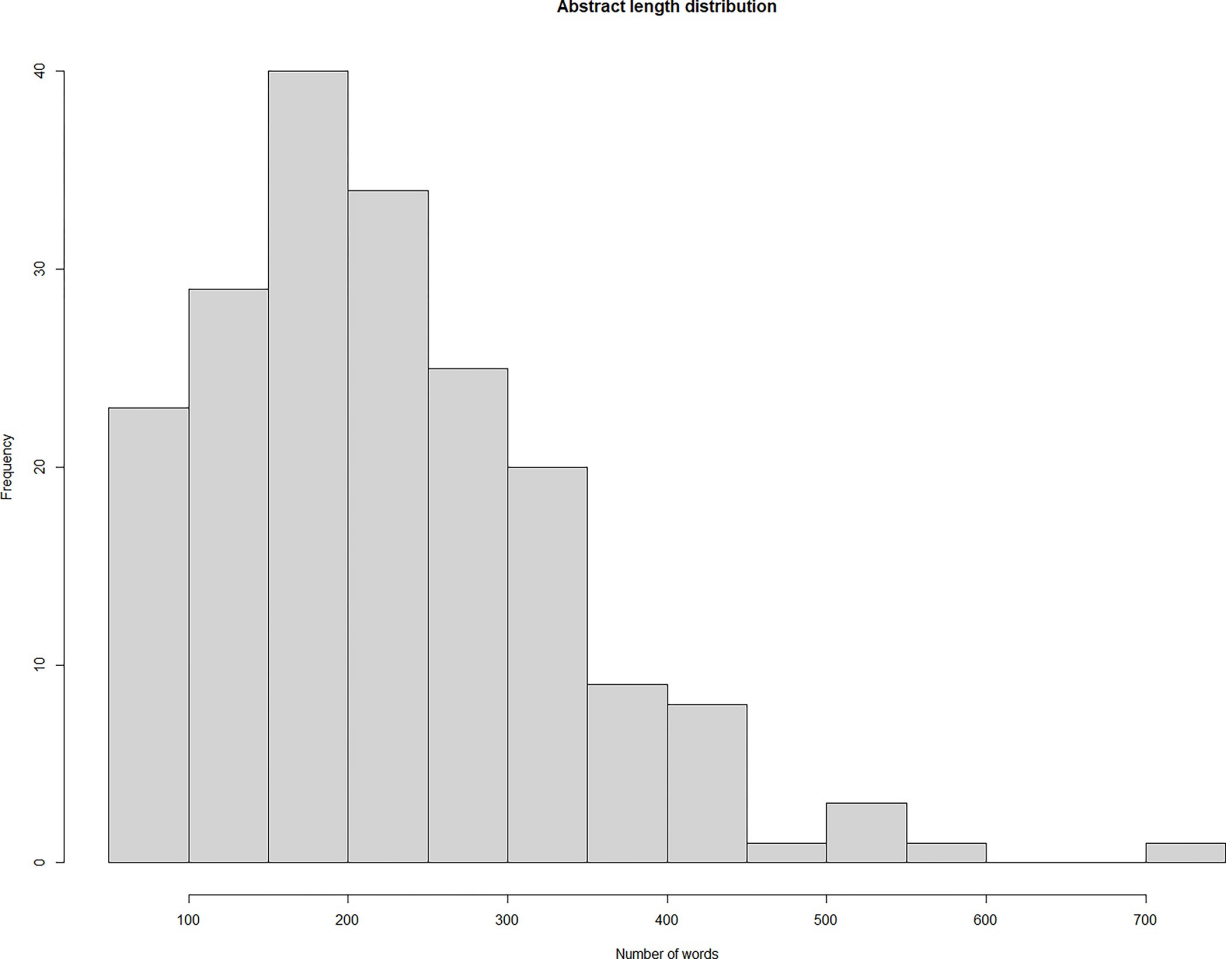
A histogram of project-abstract length, measured in word count.

Once the list of all 220 projects and their team members was compiled, a separate search was performed to collect textual descriptions for each project via several channels: (a) the official website of an organization involved in the project, (b) newspaper articles, or (c) the website of the funding entity. When no information about the collaboration was available online, we requested a brief description of activities from the principal investigator of the leading organization. Thus, our final sample consists of 194 R&D projects with a description longer than 50 words. This represents 88% of the entire list of funded projects in the time period of study. For the remaining 12% we were either unable to obtain a textual description or the one we had was too short and therefore insufficient to carry out a meaningful textual analysis. Most project descriptions mention the objective of the partnership, planned activities, and expected results. As shown in Fig 1, the majority of abstract lengths fall within a narrow range of 50 to 450 words, with only a few outliers. Since the mean (234 words) and the median (221 words) values are rather close, we can assert that the abstracts in our sample are of comparable length.

The resulting R&D network consists of 362 individual organizations. 78% of them are private for-profits and about a third of all entities (nodes) participated in more than one project. The total number of realized links (edges) is 779, and roughly 5% of them were repeated at least once in the 7-year period. Table 1 provides descriptive statistics of the final sample, on which the analysis was performed.

**Table 1:** Descriptive statistics of the final sample.

**Total number of collaborative projects**	**194**
Min project size (number of partners)	2
Max project size	6
Average project size	3.2
**Total number of organizations**	**362**
Associations	7
Health research institutes	6
Private for-profits	282
Technological institutes	9
Research institutes	5
University departments and university-affiliated research centers	44
Others	9

The “Technological institutes” of Valencia can be considered a unique element of the local innovation ecosystem, and as such merit further contextualization. Established with support from regional business associations and the government between the 1970s and the 1990s, the institutes operate as private research non-profit entities, whose primary goal is to support regional SMEs in advancing their capacities and innovative activity. Each institute is housed in a single geographic location (i.e., no dispersion of research activity), and is generally dedicated to a specific field, such as energy, textile, biomechanics, or others. The region also has several independent research centers, which do not belong to a university structure. Others work exclusively on health-related topics. These are the so-called “Health research institutes” (see Table 1).

Finally, with respect to university-type beneficiaries, we have disaggregated the larger organizations into specific departments and teams. This means that every time a grant resolution referred to a particular university, a manual search was performed to identify the exact entity within the university structure that engaged in the collaboration. This allows us to build a fine-grained image of the regional R&D network and more importantly–it facilitates the operationalization of repeated engagements. Take for instance the following scenario: a company *x* completing two projects with university *y* can hardly be considered a case of repeated engagement, unless we can confirm that both instances concerned the same department or research team within the university (disaggregate level). More details on the operationalization of all variables are provided in the following section.

## 4. Variables and methods

**Fig 2:**
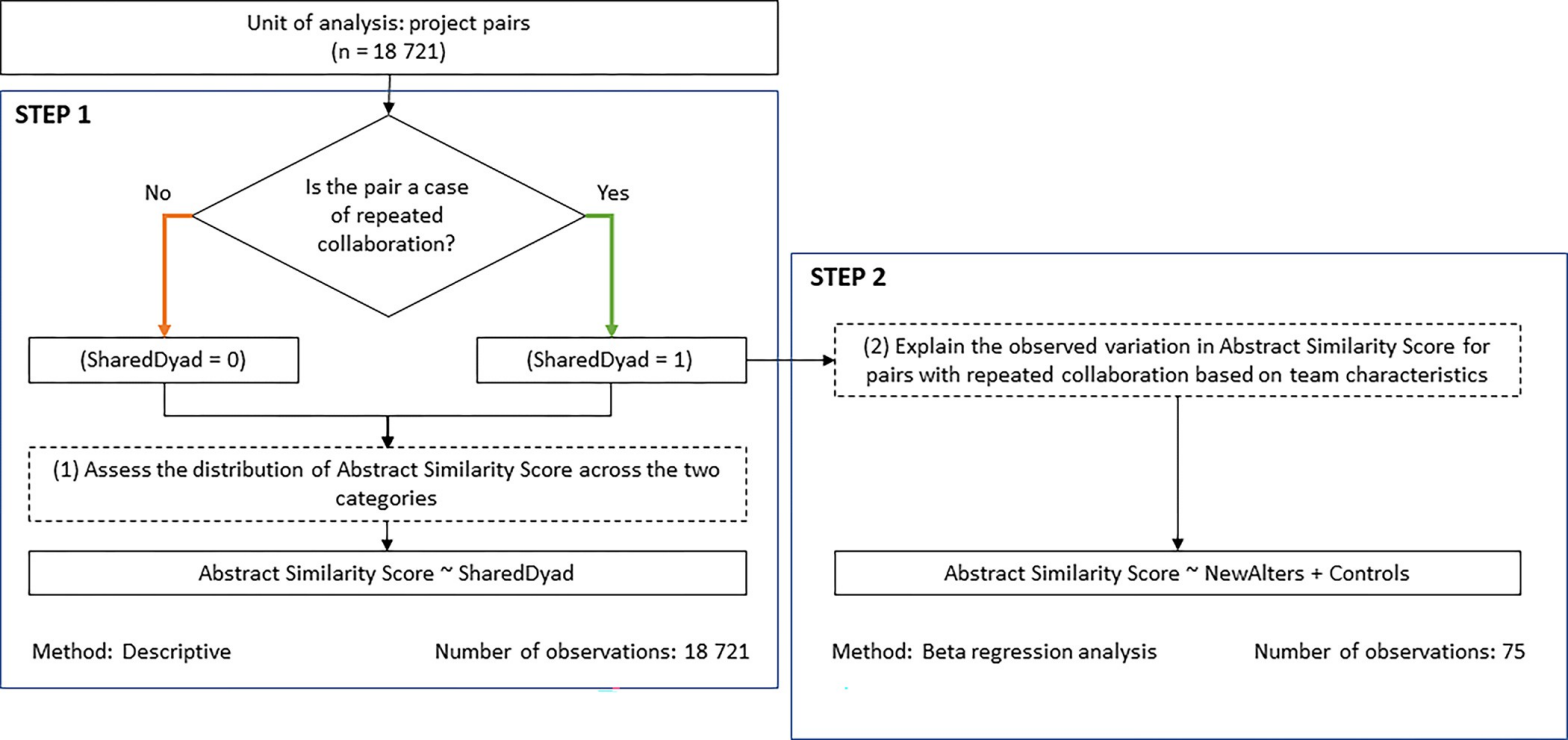
A graphical representation of the 2-step methodological approach.

To answer the main research questions, we build a 2-step approach, and our unit of analysis is the pair of R&D projects. First, we construct all possible combinations of project pairs. Since our sample consists of n = 194 projects, all pairs amount to N = n*(n-1)/2 = 18721. Then we compare those that share a common dyad of partners–what we consider instances of repeated collaboration–to those that do not. We use descriptive analysis to shed light on the first research question. In the second stage, we isolate only project pairs which represent instances of repeated collaboration, meaning: they share at least one partner dyad in common (75 pairs), in order to test how the access to diverse sources of knowledge and resources of the two organizations influences the observed thematic orientation in their repeated engagements (explorative vs exploitative). In this second stage, we run a beta regression model, which is particularly suitable when the variable of interest is continuous and restricted to the interval (0,1) [59]. Fig 2 summarizes the 2-step methodological approach graphically. Below we elaborate the operationalization of our dependent and independent variables.

### 4.1. Constructing a measure of ties’ thematic orientation

Since we are interested in analyzing whether organizations leverage repeated R&D collaborations to further exploit a particular topic or explore new ones, our primary dependent variable compares the thematic similarity between pairs of projects. Let us first revise the logic of this approach before diving into the empirical calculation.

Following the “connectionist” view of inter-organizational ties as pipelines that transmit tangible and intangible resources [20,23], one arguably reliable way to infer the content of those “pipe” flows is by tracing the description provided by the *actors themselves*. Joint project abstracts, or other types of descriptive project documentation, tend to provide sufficient information on–among other things–the specific area of intervention that partner organizations are focusing their collaboration on. Analyzing large volumes of text, however, is both challenging and burdensome. Fortunately, recent advancements in machine learning and NLP have opened up new possibilities for systematic interpretation of textual documents, including thematic classification and comparison.

Instances of NLP application in the innovation and management literature have proliferated, but so far they focus primarily on textual data from patents [60]. Balsmeier et al., for example, introduce a measure of patent novelty, based on the first occurrence of a word in the patent corpus [34]. Kaplan & Vakili use topic modelling, an unsupervised machine learning technique, to uncover the emergence of new topics in patent data and interpret those as cognitive breakthroughs [35]. Also relying on textual analysis, Kelly et al. construct a measure of lexical similarity to quantify commonality in the topical content of patents, in order to identify significant ones–that is patents whose content is distinct from prior patents (more novel), but similar to future ones (more impactful) [36].

Here we propose to leverage some of these advancements to measure the lexical similarity between pairs of R&D project abstracts so as to discern if repeated collaborations deal with the same topic. Lexical similarity is determined by the degree of lexical overlap, that is: how many terms from document *i* also appear in document *j*. It is a corpus-based method, which takes into account the co-occurrence of words across the entire collection of documents (corpus) [61,62]. We assume that projects whose descriptions show high levels of lexical similarity represent collaborative work in thematically proximate fields. When those projects were carried out by the same teams, we can interpret their repeated engagements as a continuation of previous work (thematic exploitation). Alternatively, lower similarity between project descriptions suggests that partners likely explored a completely different topic in their subsequent R&D collaboration.

The following section details all steps in the calculation of the Abstract Similarity Score.

#### 4.1.1. Measuring abstract similarity

Based on the sample of 194 regional collaborative R&D projects, we calculate a lexical similarity score for all possible pairs of project abstracts (18721 pairs). We begin by constructing a document-term matrix (DTM), whereby each row represents a unique document (project abstract) and each column represents one term (word). The value of each matrix cell *ij* reflects the number of times term *j* appears in document *i*. Before creating the DTM, the corpus of abstracts is pre-processed. This includes translating project abstracts from Spanish to English, and removing punctuation, numbers and stopwords, such as pronouns, articles, specific verbs, and other common speech elements which carry little useful information. In addition, terms are trimmed to their meaningful linguistic base or root form, called a lemma. This yields a total of 3846 unique terms. A full account of the pre-processing steps is available in the section Supporting Information (see S1 Fig).

As highlighted by Kelly et al., a key consideration in building any similarity metric for a pair of text documents is to appropriately weigh the words by their importance [36]. This is particularly crucial for our sample, since project descriptions follow a common structure and certain words (“objective”, “results”, “activities”) will be registered with greater frequency across the majority of text pairs, making them appear more similar than they really are. To account for that, we employ the “term-frequency-inverse-document-frequency” (TF-IDF) transformation method.

This method weights the registered occurrence of term *j* in document *i*, relative to its occurrence in the entire collection of documents *N*_*i*_ [63]. Consider the following eq:

$$W_{ij}={TF}_{ij}*{IDF}_{j}=\left(\frac{n_{ij}}{l_{i}}\right)*\left(log\frac{N_{i}}{N_{ij}}\right)$$ (1)

where *n*_*ij*_ is the number of times word *j* appears in document *i*, *l*_*i*_ the length of *i* in terms of total number of words, *N*_*i*_ is the total number of documents in the corpus, while *N*_*ij*_ is the number of documents in which term *j* appears. The terms with higher *W*_*ij*_ will be those that appear relatively often within a document, but do not appear in the rest of the corpus. These terms are therefore more representative of the document’s semantic content. Put differently, the TF-IDF approach allows us to overweight words which improve the diagnostic of an abstract´s topical content [36].

The final DTM (dimensions: 194 x 3846) is quite sparse, and most term-frequency vectors contain many 0 values. To estimate how textually close two project abstracts are, we use cosine similarity, which is measured by the cosine of the angle between a pair of term-frequency vectors and determines whether they are roughly pointing in the same direction. It is one of the earliest and most widely used distributional measures [64]. The advantage of using cosine similarity is that it ignores zero-matches, essentially safeguarding against false positives. For example, two term-frequency vectors may have many 0 values in common, but this does not make them similar, since the corresponding documents share few words. Cosine similarity focuses on the words two vectors have in common and the respective weight of these words [65]. It is a continuous metric that goes from 0 to 1. A high similarity score implies that two abstracts use the same set of words in the same proportion, while a lower similarity value shows no significant overlap between the texts.

#### 4.1.2. Illustrating the method with practical examples

Next, we discuss the meaning of the Abstract Similarity Score in practice. We examine first a pair of projects, which has one of the highest similarity scores (0.44) in our sample. Given that abstract length surpasses 300 words, we have included only selected excerpts from the text, which highlight succinctly what each project is about.

Project ***a*** description

*“[…] Detection and control of sulphate-reducing bacteria in drinking water infrastructures is presented in order to detect the critical points of the drinking water distribution network and implement the necessary improvements to reduce the risk of leaks and prevent water from losing its quality*. *The main objective of the project is to control and eliminate the development of sulphate-reducing bacteria in drinking water infrastructures through the development of new techniques for the detection of microorganisms and the functionalization of surfaces*, *reducing the risk of breaks and leaks and increasing the resilience of the drinking water distribution system*.*”*

Project ***b*** description

*“[…] Optimization of the hydraulic performance of the drinking water network by means of optical fibre with the aim of detecting possible leaks generated in the supply network*, *as well as locating them throughout the system*. *The main objective of the project is the development of a system for detecting leaks and structural failures in drinking water pipes*, *accurate and economical*, *operating continuously*, *based on photonic technologies*, *and more specifically on "Distributed Acoustic Sensing" (DAS)*, *which can be implemented in pipes in service and that its installation serves as a primary structural element for the implementation of future fiber optic sensors*, *specifically of water quality*, *without the need for new wiring*.*”*

From the excerpts we can see that the two projects rely on different technologies, but the area of intervention is clearly similar: improving the resilience of the drinking water distribution system. Next, we compare a second pair of projects with a low similarity score (0.02), selected at random.

Project ***a*** description

*“The main objective of this project is the research and development of an intelligent tool for dermatological exploration that assists in the detection and delimitation of the main types of skin cancer and does so in real time without the need for biopsy and through an automated and contactless technique […]”*

Project ***b*** description

*“The aim of this project is […] to improve the management of artificial wetlands for wastewater treatment*, *to naturalize their effluents*, *to minimize the impact on the receiving aquatic environment and to contribute to the mitigation of climate change […]”*

Clearly, the two projects deal with two very distinct topics, and the algorithm is accurately assigning a low similarity score to this particular pair. Note that the cosine similarity method itself does not tell us if project partners employ similar technologies, as such kind of information would be difficult to extract and requires more detailed textual data. Nevertheless, we consider that the project abstracts we work with are sufficiently descriptive to allow for meaningful comparisons of topical content.

### 4.2. Independent variables

**Fig 3:**
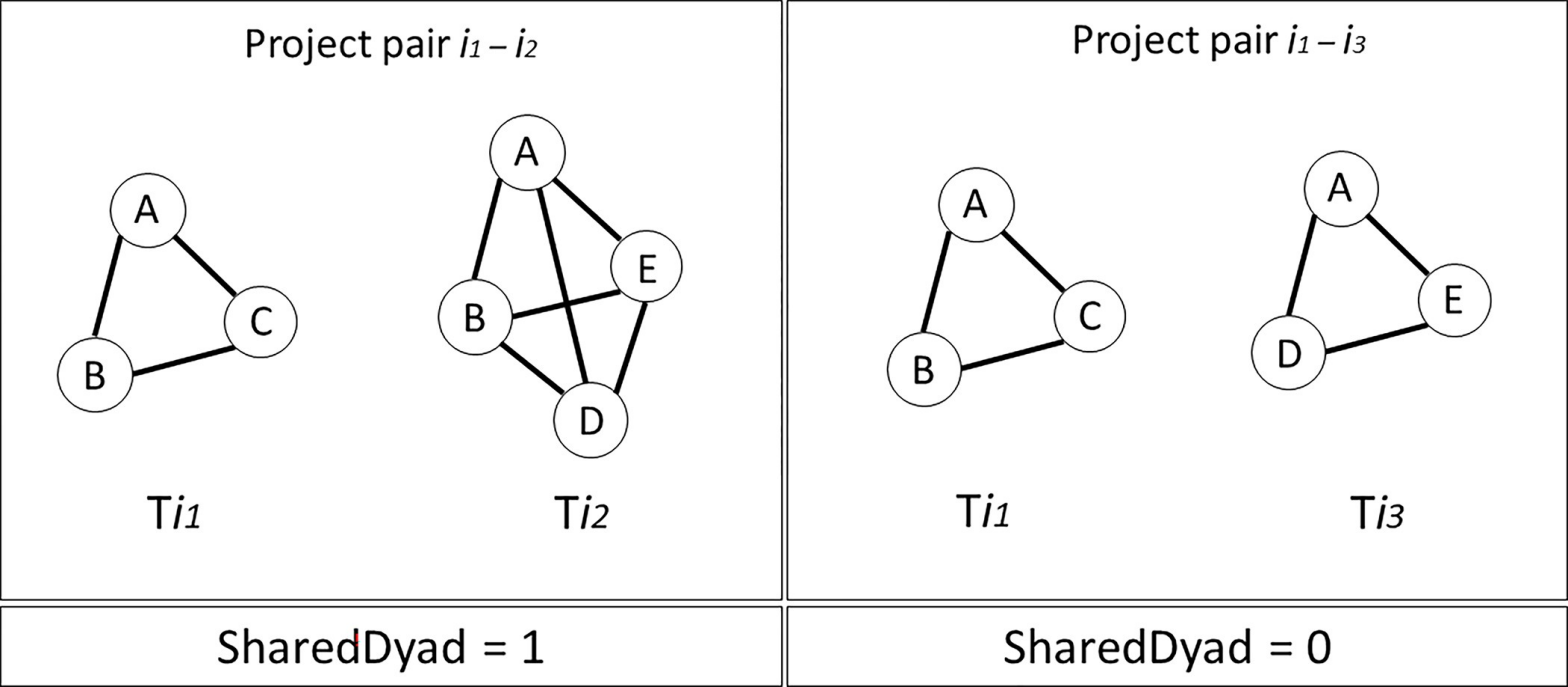
An illustrative example of the SharedDyad variable.

In the first stage of the analysis, where we want to check the similarity of projects in cases of repeated collaborations, we introduce a dummy variable, called **SharedDyad**, which is equal to 1 if the two project teams have *at least 2* organizations in common. In other words, for a pair of project abstracts *i*_*1*_*-i*_*2*_, we compare the team of partners T_i1_ to the team of partners T_i2_. Assume that project *i*_*1*_ was carried out by T_i1_ = [A, B, C], while project *i*_*2*_ was executed by T_i2_ = [A, B, D, E], where A, B, C, D and E are five unique organizations. Since the tie between A and B has persisted in both projects, the variable **SharedDyad** would assume the value of 1 even though the two teams are not completely identical and contain additional partners [E, C and D]. **SharedDyad** is equal to 0 when the two teams have only 1 or no partners in common. This approach is consistent with other studies on team recurrence, which also set a minimum threshold of one repetition to qualify strong ties [66,67]. Fig 3 provides an illustrated example to further clarify the operationalization of **SharedDyad**. We opted for a dichotomous variable, rather than a categorical one, because in our sample instances of 3 shared partners were extremely rare. Therefore, we cannot make a strong distinction between repeated dyads vs. repeated triads, but we believe that comparing the two cases may yield interesting insights.

In the second stage of the analysis, where we concentrate exclusively on repeated collaborations with **SharedDyad** = 1, we want to test how the joint access to diverse knowledge and resources for recurring partners influences the observed thematic orientation (exploitative vs explorative) of their repeated engagements.

Our primary explanatory variable is thus the social capital of both partners, accrued in the time between the first and the second collaboration and reflected in the measure **NewAlters. NewAlters** is a continuous variable which for each pair of organizations A-B counts how many *new distinct* entities did A and B connect to since their first engagement, excluding any of the partners in the projects where A and B jointly participate. Note that we only consider first-order direct connections. While indirect links may also benefit the recipient’s knowledge production, it is direct relationships that collect and process the indirect information and deliver it to the focal node, or in our case–the pair of nodes [24]. The assumption here is that the total number of unique pipelines A and B can draw upon for external knowledge will influence the extent to which the pair may explore new topics when re-engaging again.

We also introduce several controls. First, we construct a dummy variable **ExtraPartners,** which takes the value of 1 when at least one of the repeated collaborations involved additional team members. This means that unless both projects *i*_*1*_ and *i*_*2*_ were carried out exclusively by the same pair of organizations, **ExtraPartners** will be equal to 1. Assuming that extra partners can bring in a unique set of knowledge to the collaboration, their presence in the consortium can reasonably influence the degree of thematic exploration in repeated engagements.

**Fig 4:**
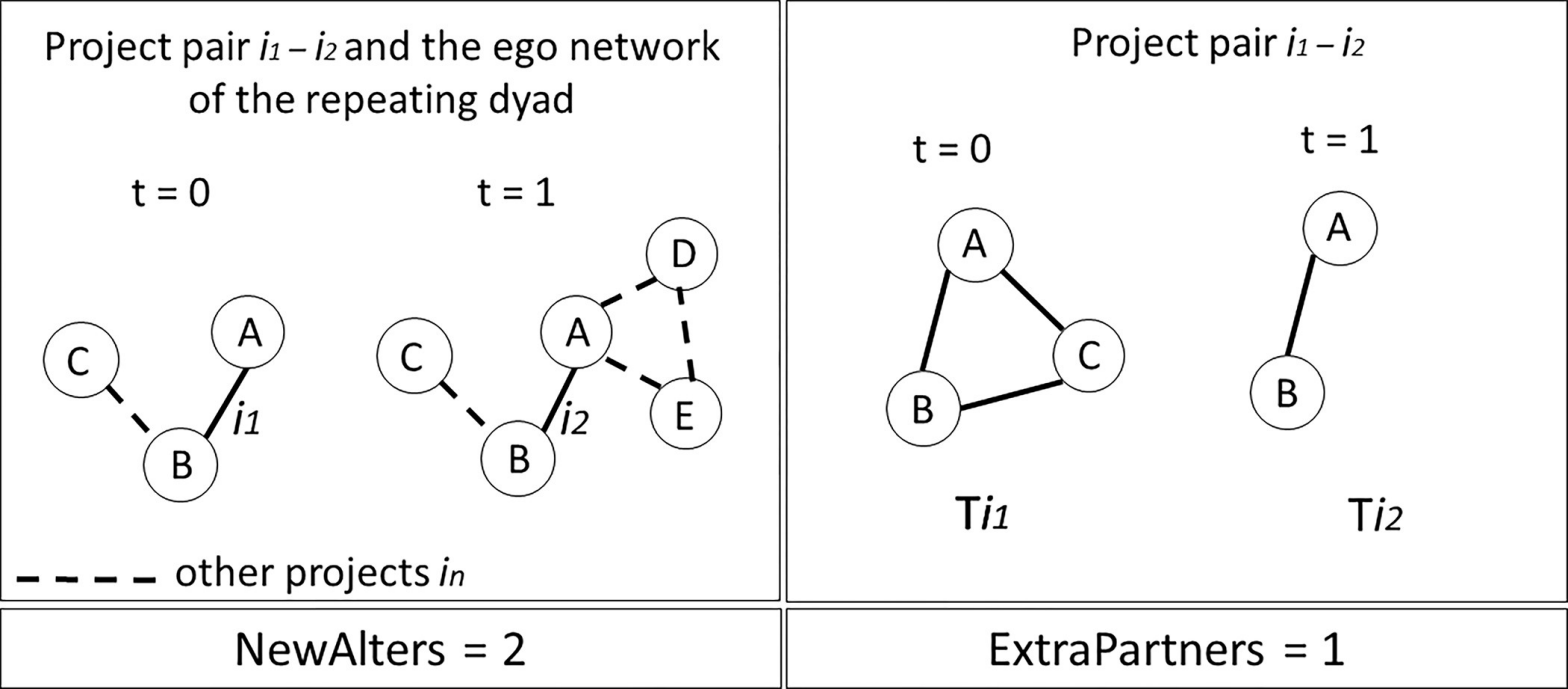
An illustrative example of the NewAlters and ExtraPartners variables.

Fig 4 illustrates the operationalization of **NewAlters** and **ExtraPartners,** using concrete examples. In the case of **NewAlters**, we can see that at the time of the second collaboration between A and B (at t = 1), the pair is connected to 2 new organizations [D, E], to whom neither A nor B had a connection at t = 0. Therefore, in the example provided **NewAlters** is equal to 2. In the case of **ExtraPartners** in Fig 4, since one of the collaborations between A and B involves an additional partner C, the dummy variable takes the value of 1.

We further control for the institutional characteristics of the two partners in the shared dyad. If the pair involves one public research organization (PRO) (technological institute, university-affiliated or independent research center) and a firm, **PRO-Firm** takes the value of 1, and 0 otherwise. If both members of the shared dyad are PROs, the dummy variable **PRO-PRO** takes the value of 1, and 0 otherwise. This allows us to distinguish between the behavior of a firm re-engaging with a PRO, as opposed to two PROs collaborating again. Finally, we also control for the time lag between the first and second collaboration. Since the public calls are launched on an annual basis, the variable **TimeLag** is a simple count of the number of years passed between the first and second engagement. The model to be estimated is given by:

Abstract Similarity Score = NewAlters + ExtraPartners + PRO-Firm + PRO-PRO + TimeLag

## 5. Results and discussion

In this section we present the results of the two-stage analysis.

### 5.1. Repeated engagement and thematic orientation: Descriptive results

We begin by exploring the distribution of Abstract Similarity Score and SharedDyad (Table 2). One immediate observation is that the majority of abstract pairs show no significant overlap in textual content. The distribution is highly skewed, with only a small fraction of pairs being very closely related. On average, abstract pairs have a lexical similarity of 0.029. This is not surprising since the open calls we considered target R&D collaborations from a range of sectors and are very thematically diverse. As for team pairs, we can see that a small fraction of project pairs contains a recurrent dyad of partners (i.e., 75 out of 18721, or 0.4%).

**Table 2:** Descriptive statistics for variables Abstract Similarity Score and SharedDyad.

Statistic	N	Mean	St. Dev.	Min	Max
Abstract Similarity Score	18 721	0.029	0.030	0.000	0.673
SharedDyad	18 721	0.004	0.063	0.000	1.000

**Fig 5:**
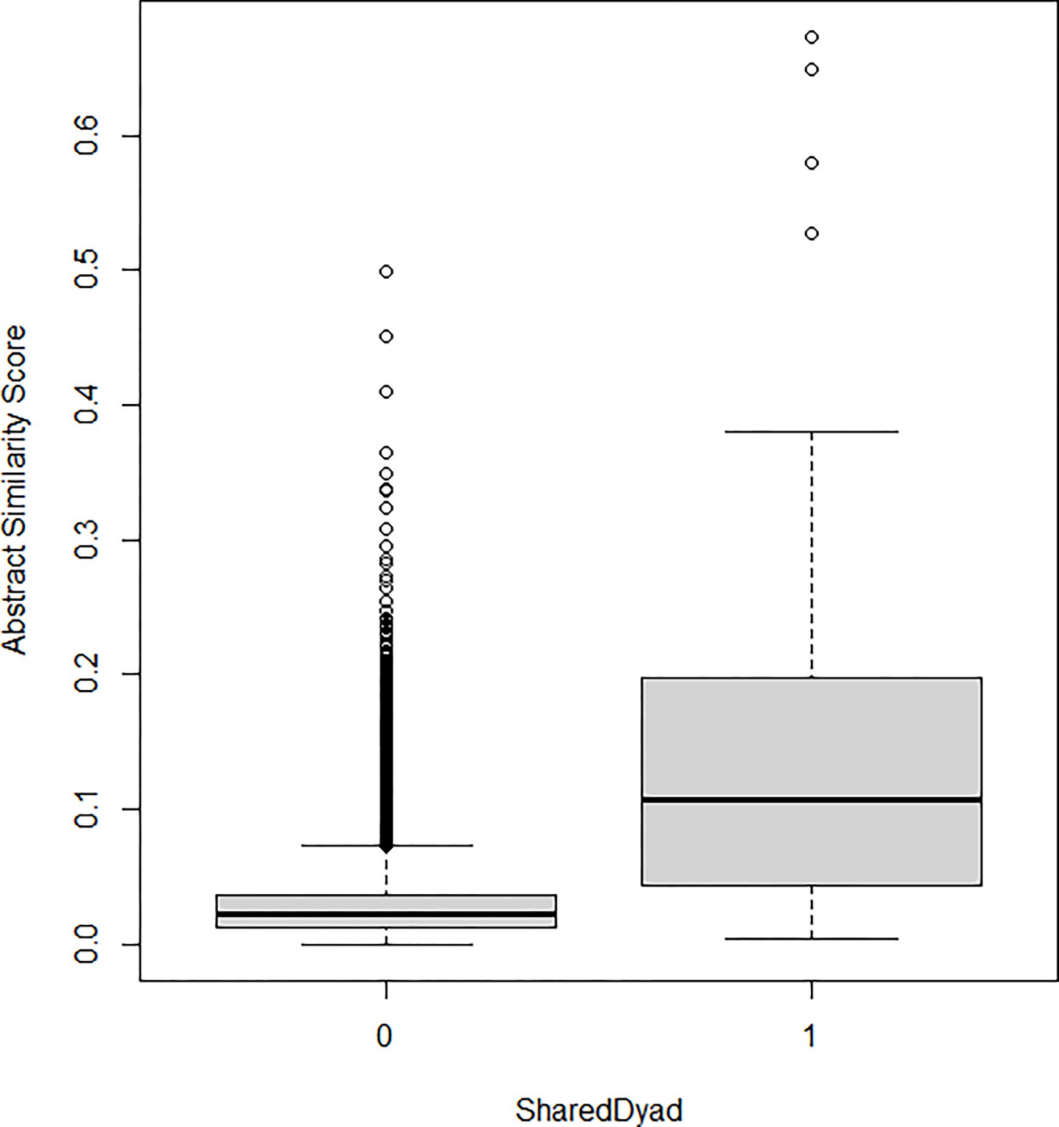
A boxplot, comparing instances of repeated collaborations (SharedDyad = 1) to the rest of project pairs (SharedDyad = 0).

The two variables are positively correlated. Since one of them is dichotomous, we apply a special case of the Pearson correlation coefficient, namely the Point-Biserial correlation coefficient. Its calculated value is 0.26, significant at the 1%. We also explore the distribution of the Abstract Similarity Score for pairs of projects with no dyad vs. those with at least 1 dyad in common. Fig 5 shows the resulting plot.

What is visible from Fig 5 is that, on the whole, project pairs which have at least one repeating pair of partners (a shared dyad), show higher median scores of thematic similarity than projects which share only one or no partners at all. However, this generally positive relationship between repeated engagements and abstract similarity is far from straight-forward. In fact, we observe a great degree of variation across project pairs with a shared dyad. The interquartile range, which accounts for the middle 50% of scores, goes between 0.05 and 0.2, while the maximum abstract similarity score (excluding outliers) is as high as 0.4. At the same time, the bar on the left-hand side contains multiple outliers: pairs of projects which exhibit relatively high similarity but were carried out by completely different teams. This may be attributed to unobserved geographical proximity (i.e. organizations belong to the same cluster and therefore work on similar topics) or the presence of a common third-party, which links two distinct consortia (triadic closure) [68]. Hence, in response to our first research question, we find that repeated collaborations are not univocally associated with topic exploitation and other factors seem to influence the thematic orientation of strong ties. It appears that in some instances, collaborating pairs use subsequent R&D partnerships to extend prior work along the same topic, adopting an exploitative approach, while others do not. This provides original support to the argument put forward in the theoretical section, namely that inter-organizational network links are far from homogeneous and that only some, but not all, instances of repeated coupling between actors are associated with the exploitation of the same research topic. This implies that “getting caught up” in one type of activity after several collaborations may not necessarily be a product of the structural setting alone and the existence of strong coupling, as much as it is a product of organizations’ strategic choices about how they use their strong ties.

**Fig 6:**
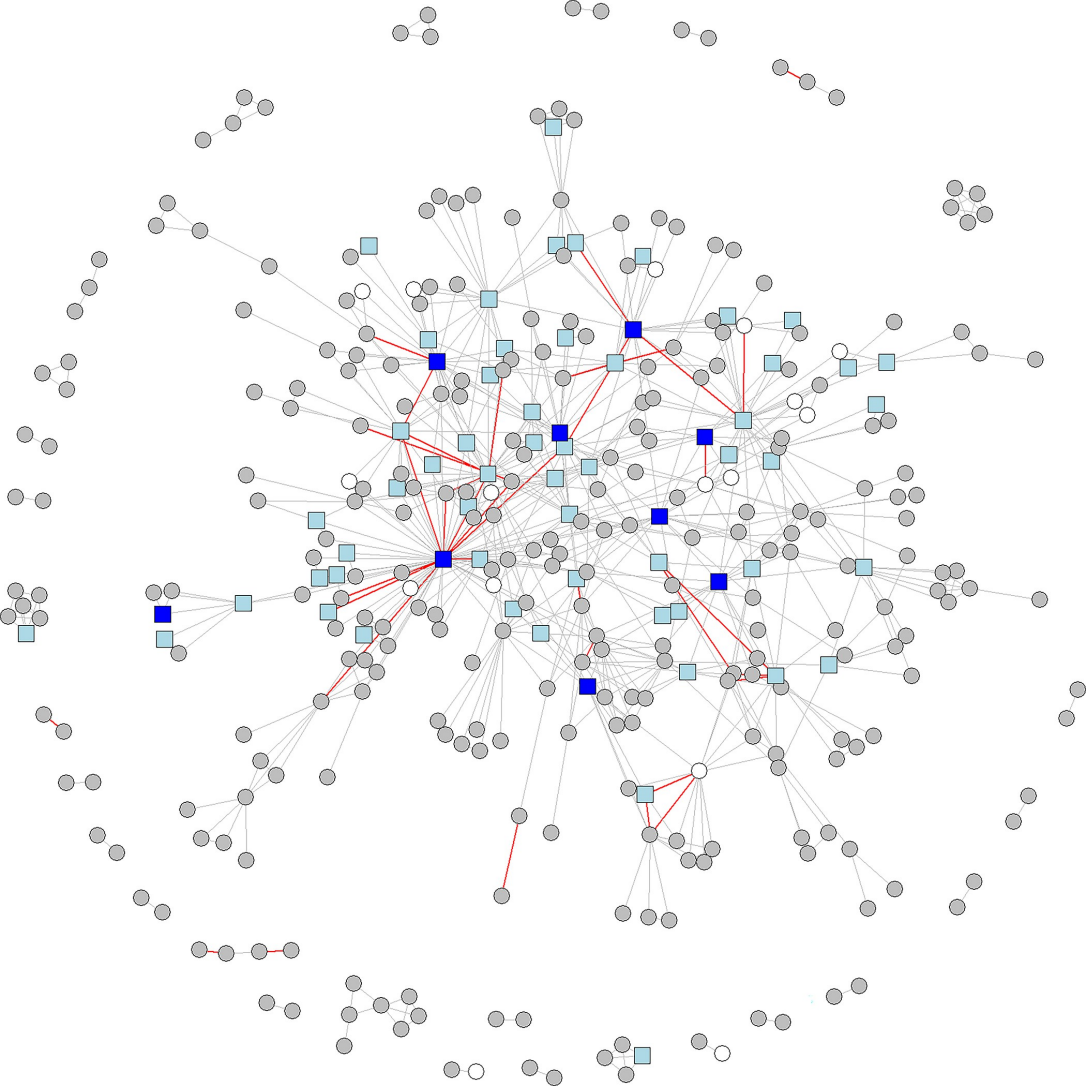
Aspatial map of the R&D network (2016–2022). Repeated ties are marked in red. Nodes legend: grey circle (firm), dark blue square (technological institute), light blue square (independent or university-affiliated research center), white circle (other).

Fig 6 shows an aspatial map of the R&D network and highlights the structural location of all repeated ties.

The resulting network appears centralized around the main technological institutes and several other PROs. This is consistent with studies of regional and extra-regional networks, where PROs were found to serve as intermediaries, and thus appear as frequent partners in regional collaborations [69,70].

Although instances of repeated ties are relatively scarce, they appear both in the core and in the periphery. Given the positive effect of strong bonds on inter-organizational trust, their “balanced” distribution is beneficial for the flow of tacit complex knowledge across the network architecture. Fig 6 also showcases the institutional heterogeneity of actors involved in recurrent collaborations, which further motivates the second part of our analysis, where we explore how a dyad’s access to new distinct alters may moderate the displayed thematic orientation (explorative vs exploitative) of repeated ties.

### 5.2. The role of partners’ social capital

In this section, we concentrate exclusively on pairs of projects which have at least one repeated dyad (in total 75 pairs). We begin by presenting descriptive statistics (Table 3) followed by a Point-Biserial correlation matrix (Table 4), chosen for its applicability to data that include both dichotomous and continuous variables.

**Table 3:** Descriptive statistics for the main variables employed in the beta regression analysis.

Variable	N	Mean	Median	St. Dev.	Min	Max
Abstract Similarity Score	75	0.153	0.107	0.148	0.004	0.673
NewAlters	75	11.387	4	15.861	0	64
ExtraPartners	75	0.720	1	0.452	0	1
PRO-Firm	75	0.267	0	0.445	0	1
PRO-PRO	75	0.320	0	0.470	0	1
TimeLag	75	1.560	1	1.255	0	6

**Table 4:** Point-Biserial correlation matrix between the main dependent variable (Abstract Similarity Score) and the explanatory variables.

Parameter	NewAlters	ExtraPartners	PRO-Firm	PRO-PRO	TimeLag
**Abstract Similarity Score**	-0.39**	-0.47***	-0.24	-0.34*	0.11
**NewAlters**		0.39**	0.24	0.34*	0.18
**ExtraPartners**			0.38**	0.43**	-0.03
**PRO-Firm**				-0.41**	-0.004
**PRO-PRO**					-0.22

The high mean value of ExtraPartners implies that for most project pairs, the two repeating partners were not the only members in the consortium. The values for NewAlters vary between 0 and 64. This suggests that in some cases, the repeating partners built an extensive network of direct relationships after the first collaboration and by the time of executing the second one, the dyad had collectively accumulated 68 new unique alters in their ego network. The average time lag between the two collaborations is 1.6 years.

Examining the matrix of correlations, we note that the number of new alters a pair of partners gains, is negatively correlated with the observed Abstract Similarity Score, implying that greater social capital is positively associated with exploration of distinct research topics. Furthermore, most project pairs, where the repeated dyad is embedded in a rich network of alters, also include extra partners in the consortium. Connectivity of the repeated dyad seems to correlate positively with certain institutional characteristics of the organizations. This can be expected since PROs tend to have a disproportionately high degree centrality. They establish numerous links with other nodes, and have sufficient human, administrative and financial capacity to maintain them [71].

Table 5 shows the results of the beta regression. We first run a base Model 0, where we include only controls, followed by Model 1 including only the primary explanatory variable and a third model where all relevant variables are featured.

**Table 5:** Results of the beta regression, assessing the effect of NewAlters on the Abstract Similarity Score.

	Dependent variable: Abstract Similarity Score		
	(Model 0)	(Model 1)	(Model 2)
NewAlters		-0.023*** (0.007)	-0.016*** (0.008)
ExtraPartners	0.008 (0.291)		-0.064 (0.288)
PRO-Firm	-0.883*** (0.313)		-0.517 (0.341)
PRO-PRO	-0.863*** (0.311)		-0.491 (0.355)
TimeLag	0.050 (0.075)		0.107 (0.078)
Constant	-1.319*** (0.207)	-1.454*** (0.123)	-1.397*** (0.206)
Observations	75	75	75
Adjusted R2	0.188	0.176	0.253
Log Likelihood	74.804	71.414	76.822

Note

*p<0.1

**p<0.05

***p<0.01 Robust standard errors appear in brackets.

Both control variables, reflecting the presence of one (PRO-Firm) or two (PRO-PRO) public research organizations in the dyad, have a negative coefficient, which is significant in Model 0 but not in Model 2, suggesting that PROs are generally associated with a more exploratory strategy in repeated ties. Similarly, the coefficient for NewAlters is negative and remains significant when controlling for the institutional heterogeneity of organizations (Model 2). This means that organizations which built an extensive network of connections are also more likely to explore a different topic when re-engaging with a previous partner. This behavior can be attributed to their enhanced access to new knowledge and ideas, which is likely to inspire greater creativity and the exploration of diverse research avenues. This rationale is grounded in the principles of social network theory. Connectivity facilitates the flow of information, which benefits the knowledge base of the organization, but also equips it with the capacity to identify new research trajectories. Conversely, organizations which lack sufficient network connectivity, may have limited access to novel knowledge and ideas, and will therefore be more inclined to adopt an exploitation strategy when engaging repeatedly with the same partner. Our additional control variable ExtraPartners and TimeLag do not seem to exert a significant influence on the thematic orientation of strong ties.

The results of the regression analysis suggest that the range of two partners’ combined ego network favors the exploration of new research trajectories in recurrent collaborations. The social capital available to the pair appears to promote access to diverse sources of knowledge and facilitate the shift from old research trajectories into new ones. In other words, when organizations with high number of new alters build strong ties, these ties exhibit more topic diversity, than strong links between isolated nodes with little connection to the rest of the network. Because of the relatively high correlation between NewAlters and PRO-PRO, and the fact that most central actors tend to be PROs, we cannot unequivocally attribute the “diversifying” effect to one factor alone.

On a theoretical level, the results showcase that social capital embedded in a particular linkage cannot be treated as a static asset. If one or both of the participating organizations in the dyad has a rich network of external contacts and is capable of renewing its knowledge base over time, the value of the established contact may persist longer and cycles of topic exploitation can be followed by the exploration of new research avenues. In other words, the value of strong ties may not necessarily “wear off” in an inverted U-shape the way conventional theory suggests. Moreover, these findings have implications for the framing of the proximity paradox, which seems to consider dyadic relations in isolation of the surrounding environment. When two organizations build strong ties, they do not automatically detach themselves from third parties. The conceptualization of the proximity paradox can therefore benefit from adopting a triadic approach. This will allow researchers to better understand the depreciating value of strong bonds over time. Of course, further research is needed to analyze when exactly third-party links enrich the knowledge base of a particular organization and how this influences the value of a node’s persistent ties.

## 6. Conclusion

This study aims to investigate empirically the relationship between the strength of collaborative inter-organizational ties and their thematic orientation (explorative vs exploitative) in the context of Valencia’s policy-induced R&D network. Moreover, it examines how this relationship plays out for partners with different levels of connectivity. Thus, the paper responds to recent calls for greater focus on networks’ relational aspects and interaction processes [19,21], by examining specifically how organizations approach repeated collaborations. The study delivers several important insights.

First, it demonstrates that recurring partnerships between organizations in an R&D network are not always associated with the exploitation of the same topic. Building strong bonds may also involve the exploration of new topics and the mobilization of new knowledge domains. Nevertheless, in the case of Valencia’s R&D network, the latter scenario appears more likely when the partners involved are connected to a larger network of diverse contacts between the first and second instance of collaboration and can access novel knowledge and ideas. Hence, this study offers original evidence on the heterogeneity of network ties and the importance of considering the function of strong bonds between partners, given that organizations’ approach to repeated collaborations can be evidently distinct. Empirically, this paper introduces a novel approach to measuring thematic orientation in R&D collaborations, which is based on lexical similarity of project abstracts. With regards to policymaking, the analysis is also highly relevant, especially in relation to the smart specialization paradigm, which has become a cornerstone of EU Cohesion Policy [72]. When efforts are directed toward accumulating competitive advantage in prioritized areas, building strong ties between firms should be stimulated. Conversely, if the network is experiencing stagnation, exploring new themes and mobilizing novel knowledge would be far more critical. A scenario like this calls for investment in partnerships that enroll a broader range of organizations (including PROs) with a rich network of contacts, both local and extra-regional, in order to diversify the thematic focus of R&D collaborations, and avoid deepening the focus on declining industries.

Finally, this study is not without limitations. The most significant one concerns the operationalization of our dependent variable, which builds on the lexical similarity of project abstracts. Since it is plausible that two abstracts describe the same area of research through different terminology, it would be beneficial to repeat the analysis using an advanced semantic similarity method, which is capable of interpreting the meaning of textual information. In addition, when constructing our primary independent variable for network connectivity, we consider only links to other nodes within the same network. In reality, actors may be able to access external knowledge through complementary linkages in parallel unobserved formal or informal networks. Both of these limitations offer promising avenues for further research. More importantly, we believe that doubling down on efforts to examine the nature and content of inter-organizational ties can be particularly beneficial for fleshing out the big questions surrounding the co-evolution of network structures and knowledge flow.

## Supporting information

S1 Fig A flowchart of the pre-processing sequence leading to the final document-term matrix. The analysis was performed in R using the tm (text mining) package (https://tm.r-forge.r-project.org/). The full list of English stopwords can be accessed through the tm reference manual (https://cran.r-project.org/web/packages/tm/tm.pdf). In calculating the DTM matrix, no minimum term frequency was set, meaning that all terms were included in the matrix.
(TIF)
