# Supplementary figures and images for: Does repetition equal more of the same? tie strength and thematic orientation in R&D networks
Source: PLoS One. 2024 May 23;19(5):e0303912. doi: 10.1371/journal.pone.0303912 (PMC11115229; doi:10.1371/journal.pone.0303912)

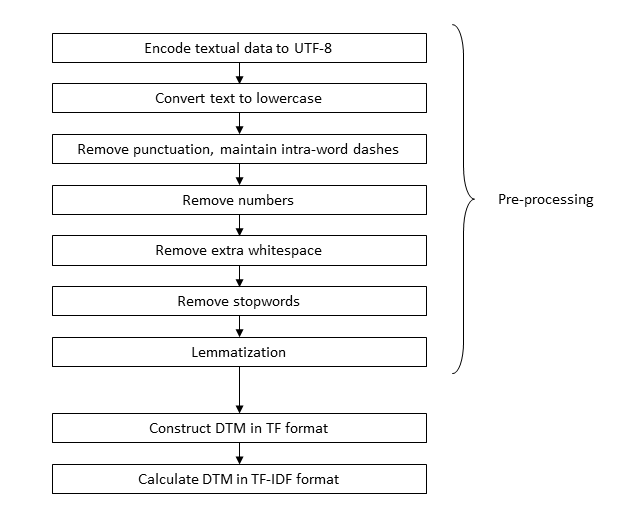

Supplement: S1 Fig — The analysis was performed in R using the tm (text mining) package (https://tm.r-forge.r-project.org/). The full list of English stopwords can be accessed through the tm reference manual (https://cran.r-project.org/web/packages/tm/tm.pdf). In calculating the DTM matrix, no minimum term frequency was set, meaning that all terms were included in the matrix. (TIF) [file pone.0303912.s001.tif]
